# Supplementary figures and images for: A Critical Review on the Use of Support Values in Tree Viewers and Bioinformatics Toolkits
Source: Mol Biol Evol. 2017 Mar 22;34(6):1535–42. doi: 10.1093/molbev/msx055 (PMC5435079; doi:10.1093/molbev/msx055)

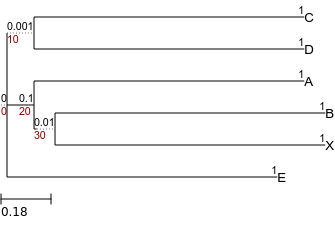

Supplement: Supplementary Data [file msx055_Supp.zip › test_cases/original_tree.png]

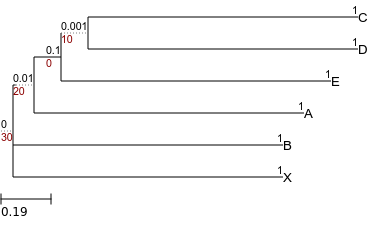

Supplement: Supplementary Data [file msx055_Supp.zip › test_cases/test_ape.sh.png]

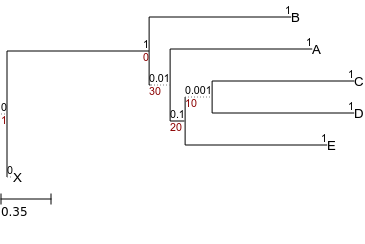

Supplement: Supplementary Data [file msx055_Supp.zip › test_cases/test_bioperl.sh.png]

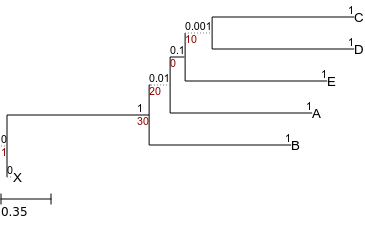

Supplement: Supplementary Data [file msx055_Supp.zip › test_cases/test_biopython.py.png]

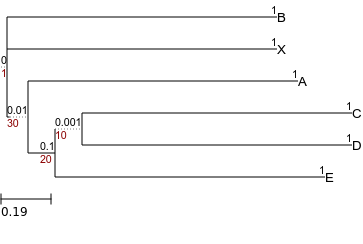

Supplement: Supplementary Data [file msx055_Supp.zip › test_cases/test_dendropy.py.png]

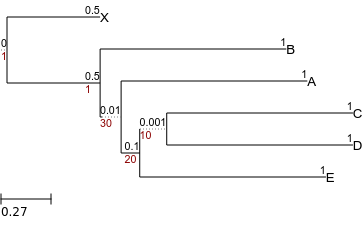

Supplement: Supplementary Data [file msx055_Supp.zip › test_cases/test_ete2.py.png]

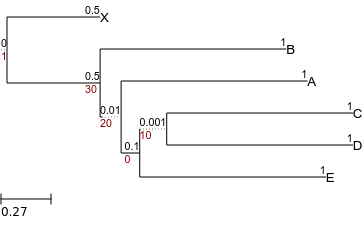

Supplement: Supplementary Data [file msx055_Supp.zip › test_cases/test_newick_utilities.sh.png]

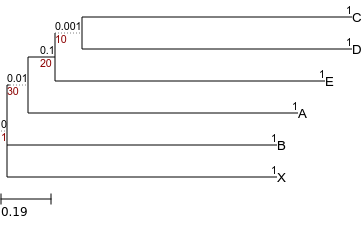

Supplement: Supplementary Data [file msx055_Supp.zip › test_cases/test_patched_ape.sh.png]
